# Blood glucose testing and primary prevention of diabetes mellitus type 2 - evaluation of the effect of evidence based patient information

**DOI:** 10.1186/1471-2458-10-15

**Published:** 2010-01-14

**Authors:** Jutta Genz, Burkhard Haastert, Gabriele Meyer, Anke Steckelberg, Hardy Müller, Frank Verheyen, Dennis Cole, Wolfgang Rathmann, Bettina Nowotny, Michael Roden, Guido Giani, Andreas Mielck, Christian Ohmann, Andrea Icks

**Affiliations:** 1 German Diabetes-Centre, Leibniz Institute at Heinrich Heine University Düsseldorf, Institute of Biometrics and Epidemiology, Auf'm Hennekamp 65, 40225 Düsseldorf, Germany; 2 Medistatistica, Lambertusweg 1b, 58809 Neuenrade, Germany; 3 Witten/Herdecke University, Faculty of Medicine, Institute of Nursing Sciences, Stockumer Straße 12, 58453 Witten, Germany; 4 University of Hamburg, MIN Faculty, Health Sciences, Hamburg, Germany; 5 WINEG - Scientific Institute for Benefit and Efficiency in Health Care, Habichtstraße 30, 22305 Hamburg, Germany; 6 German Diabetes-Centre, Leibniz Institute at Heinrich Heine University Düsseldorf, Institute of Clinical Diabetology, Auf'm Hennekamp 65, 40225 Düsseldorf, Germany; 7 Helmholtz Zentrum München - German Research Center for Environmental Health, Institute of Health Economics and Health Care Management, P.O. Box 1129, 85758 Neuherberg, Germany; 8 Heinrich-Heine-University Düsseldorf, Coordination Centre for Clinical Trials (KKS), Moorenstr. 5, 40225 Düsseldorf, Germany

## Abstract

**Background:**

Evidence-based patient information (EBPI) has been recognised as important tool for informed choice in particular in the matter of preventive options. An objective, on the best scientific evidence-based consumer information about subthreshold elevated blood glucose levels (impaired fasting glucose and impaired glucose tolerance) and primary prevention of diabetes, is not available yet. Thus we developed a web-based EBPI and aim to evaluate its effects on informed decision making in people 50 years or older.

**Methods/Design:**

We conduct a web-based randomised-controlled trial to evaluate the effect of information about elevated blood glucose levels and diabetes primary prevention on five specific outcomes: (i) knowledge of elevated blood glucose level-related issues (primary outcome); (ii) attitudes to a metabolic testing; (iii) intention to undergo a metabolic testing; (iv) decision conflict; (v) satisfaction with the information. The intervention group receives a specially developed EBPI about subthreshold elevated blood glucose levels and diabetes primary prevention, the control group information about this topic, available in the internet.

The study population consists of people between 50 and 69 years of age without known diabetes. Participants will be recruited via the internet page of the cooperating health insurance company, Techniker Krankenkasse (TK), and the internet page of the German Diabetes Centre. Outcomes will be measured through online questionnaires. We expect better informed participants in the intervention group.

**Discussion:**

The design of this study may be a prototype for other web-based prevention information and their evaluation.

**Trial registration:**

Current Controlled Trial: ISRCTN22060616.

## Background

Options for primary prevention require very careful consideration and value-neutral information, because a previously healthy population is defined as needing treatment and considered over a long period of preventive measures. Primary prevention of diabetes is much debated. Evidence-based patient information (EBPI) tailored to their needs, should be available for all consumers, because it is essential for informed choice and shared decision-making in the matter of screening procedures and preventive or treatment options [1-3]. Relating to early diagnosis, evidence-based patient information should enable the estimation of the personal risk and therefore giving a basis to the patient to estimate potential and consequences. EBPI on subthreshold elevated blood glucose levels (impaired fasting glucose, IFG, and impaired glucose tolerance, IGT) is not available yet. Since the concept of prediabetes is discussed intensively and identified of high relevance to public health we developed a web-based EBPI [4]. The development of the EBPI followed the accepted steps of EBPI development [3]: 1) systematic literature search performed by two researchers (AS and GM), 2) selection of relevant publications using predefined inclusion and exclusion criteria, 3) critical appraisal of the literature, 4) translation of the main results into information relevant for consumers using risk communication techniques, 5) and careful piloting of the EBPI within focus groups.

Subthreshold elevated blood glucose levels are characterised as impaired glucose tolerance (IGT) or impaired fasting glucose (IFG). These are risk factors for type 2 diabetes mellitus [5]. IFG and/or IGT were found in a frequency up to 25% in the older population in Germany [6]. The standardised (age and sex) incidence rates of Type 2 diabetes in this population (95% CI) per 1,000 person-years were 15.5 (12.6-19.1) in the total cohort, 20.2 (15.6-26.1) in men and 11.3 (7.9-16.1) in women [7]. Hence, a screening for elevated blood glucose levels might allow identifying a target group for primary prevention of diabetes mellitus. Randomised controlled trials in Finland and the USA suggest a reduction of diabetes mellitus diagnoses in people with subthreshold elevated blood glucose levels through lifestyle-interventions [8,9].

In this study, we aim to optimise the information on elevated blood glucose levels and diabetes primary prevention provided for people 50 years or older by a carefully developed and piloted EBPI. We aim to investigate if an EBPI could improve the decision making process.

## Methods/Design

### Development of the evidence-based patient information

The development of the EBPI followed the accepted steps of EBPI development [3]: 1) systematic literature search performed by two researchers (AS and GM), 2) selection of relevant publications using predefined inclusion and exclusion criteria, 3) critical appraisal of the literature, 4) translation of the main results into information relevant for consumers using risk communication techniques, 5) careful piloting of the EBPI within 41 participants in several focus groups.

### Study design and setting

Pilot testing covered feasibility and acceptability testing of the instruments and procedures. The testing of the instruments took place via focus groups (15 participants) and via postal evaluation (31 participants). Procedures were tested online in several passes, primarily among employees of the participating project partners. In October 2009, the information has been made available to the study population via web-based assess. We invite visitors of the internet pages of the cooperating health insurance company, Techniker Krankenkasse (TK), and the German Diabetes Centre to take part in the study. Incentives such as books and wellness funds are drawn in order to increase the response rate. Individuals who agree to participate and give their informed consent are randomly assigned to the experimental (evidence-based consumer information) or the control group (standard information). Randomisation is stratified according to sex and age.

#### Intervention

Participants are presented either internet-based EBPI (experimental group) or standard information (control group). A widely used brochure published in the internet and two articles of popular websites are used as standard information on subthreshold elevated blood glucose levels or diabetes [10-12]. The standard information offers information about prevention, early detection, sequelae and treatment of diabetes mellitus. The EBPI summarises the existing knowledge from the scientific literature on the topic.

#### Study population: Inclusion and exclusion criteria

Visitors of the above mentioned two internet pages are invited to participate and to answer online questionnaires when they are aged 50 to 69 years and without known diabetes. Participants who mention to have a diagnosis of type 2-diabetes mellitus are excluded before the statistical evaluation.

#### Study execution

**Figure 1:**
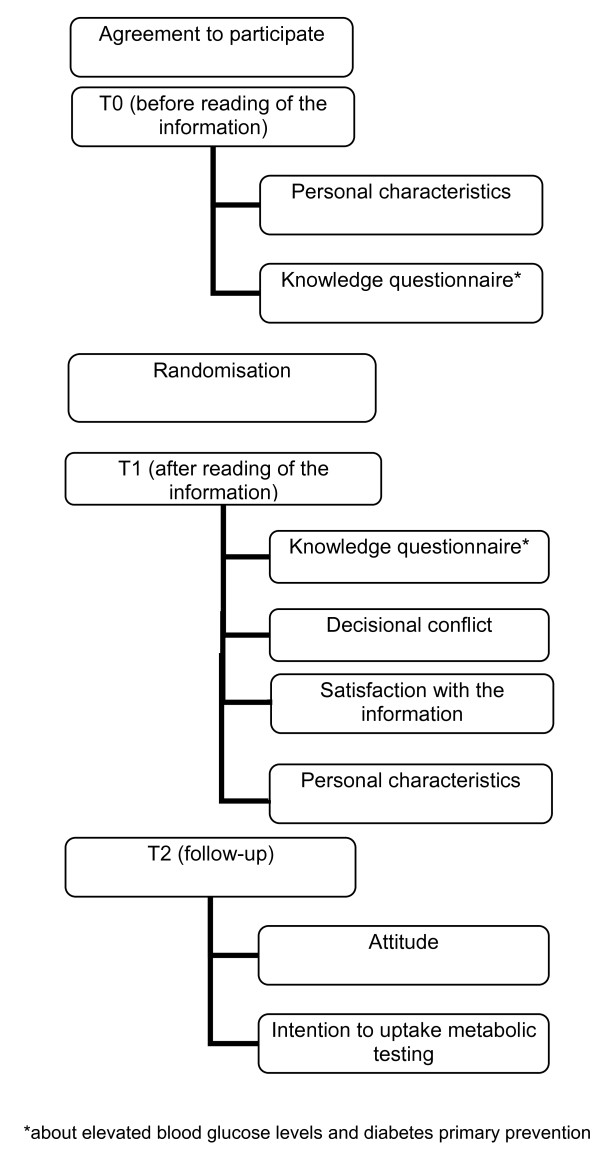
**Progress of intervention**.

Participants obtain the internet information (EBPI or standard, respectively) on blood glucose disorders, metabolic testing (diabetes-testing) and prevention strategies. The survey is carried out at three points in time (T0/T1, T2), with the first two times during one visit to the website. T0 or T1 is the questioning before or after reading the information, so that the gain of knowledge and a characterisation of later drop-outs is ascertainable. T2 is the online follow-up survey two weeks after the first online session (see Figure 1).

The variables of the T0-survey and some user behaviour, e.g. point where participants quit the study, will be recorded for later drop-out-analysis.

#### Outcomes

In this study five outcomes will be measured: (i) knowledge of elevated blood glucose level-related issues - the main outcome of the study; (ii) attitudes to a metabolic testing; (iii) intention to undergo a metabolic testing; (iv) decisional conflict; (v) satisfaction with the information. All outcomes will be assessed using self-completed online questionnaires. All translated or newly developed instruments have been carefully piloted based on accepted focus group procedures [13-17].

The main outcome 'knowledge' will be assessed using an eight-item multiple-choice-scale about the benefits and risks of blood glucose screening. Because there is no German version of a validated instrument on this topic available, we developed an own questionnaire based on Anglo-American and German instruments [18-23]. The questionnaire is adapted to the EBPI and displays the knowledge needed to make an informed choice about blood glucose testing.

Attitudes to a metabolic testing (screening test about elevated blood glucose) are determined by a four-item scale [24].

Intention to undergo metabolic testing within the next 12 months: This will be assessed using a single item question (translated into German) with a five point response scale, according to Gattellari et al 2003 [25].

Decisional conflict is measured using the decisional conflict scale (DCS). This is a 16 item scale which elicits uncertainty in choosing among health-related alternatives, contributing factors to uncertainty and the perceived effective decision making. It discriminates between subjects who make and who delay decisions. The five-point Likert scale is anchored by "strongly agree" and "strongly disagree". The DCS has met acceptable standards of reliability and validity [26,27]. A German authorised translation which has been already used in comparable studies, is available [28]. Because the measure is conducted before a decision is made, we set aside the four items which target the situation after the decision. This approach is similar to an earlier evaluation [29].

Satisfaction with the information and the information available is surveyed each with an according question using school grades.

#### Further variables

Personal data (age, sex, socio-economic variables, life satisfaction) are also assessed. A short instrument has been developed on the basis of recommendations of the German Society of Epidemiology [30]. To assess life satisfaction, a single question from the socio-economic panel is applied [31]. A scale in the form of a 10 rung ladder is used to measure subjective social status [32]. Furthermore, subjects are asked for their medical history, in particular, diabetes mellitus. Participants are also asked if they already passed a blood glucose test.

#### Randomisation

Randomisation takes place immediately before presentation of the information, deposited on a random number generator. To gain a balanced distribution of the study population characteristics, a stratified block randomisation is conducted (4 strata defined by women/men and 40-49 years/50 years and older, 1:1 randomisation between intervention and controls within each stratum using blocks of size 10).

Crossover between the study groups is not possible, because the IP-addresses of the participants are identified by the system.

#### Statistical analysis

Data of all randomised participants excluding those with self-documented diabetes will be evaluated. The primary analysis will be performed on the study population of T1 compliers with non-missing primary outcome of knowledge. The secondary analysis set will be the subset of compliers of T1 and T2.

The persons who have prematurely terminated the study without completely documented knowledge outcome of T1 will be compared with the study population on the basis of the parameters which are assessed before randomisation (age, sex, education, life satisfaction, and pre-study knowledge about elevated blood glucose), using appropriate statistical tests (chi-square test, Fisher's exact test, t-test or Wilcoxon test). Furthermore, the subgroup of the T1/T2 compliers will be compared with the study population of T1 compliers in a similar manner.

Outcomes will be compared between intervention and control group on an 'intention to treat' basis. For the description of the population in terms of relevant basic parameters - according to distribution - frequency tables, prevalences with 95% confidence intervals or mean values with standard deviations will be calculated. Scores on knowledge and quality of the decision and satisfaction will be evaluated by average scale scores with standard deviations and percentiles. The estimates will be carried out in the intervention and control group respectively in total and stratified for relevant subgroups, e.g. age (40-49 years or older) and sex. To test any effects of the intervention, statistical comparisons will be performed - according to distribution - by chi-square or Fisher's exact test, t-test or Wilcoxon's rank sum test. The tests for each target variable is to be defined before the evaluation.

The primary outcome is an ordinal score with values 0-8. The distributions between intervention and control group will be compared primarily by Wilcoxon's rank sum test. In a secondary analysis the outcome "good knowledge" is defined by dichotomisation of the ordinal knowledge score using a predefined cut point of 5 (≥ 5 versus <5). Intervention and control group will be compared by chi-square test.

The association between this primary outcome and predictors is estimated using multiple ordinal logistic regression models. A continuation-ratio logit model will be fitted [33]. To simplify the interpretation of the results the scale of the knowledge score will be reduced to 3 ordered values by the predefined classes 0-2, 3-5 and 6-8. The predictors are first examined by univariate models. Then, multiple models considering age, sex, socio-economic variables and participation in a blood glucose test will be discussed.

Then, multiple models considering age, sex, socio-economic variables, participation in a blood glucose test and pre-study knowledge about elevated blood glucose will be discussed. A final multiple regression model will be fitted.

A secondary analysis will be performed on the subpopulation of participants at T1 and T2 considering the ordinal outcomes "attitude" and "intention to undergo uptake a metabolic testing". These variables will be described non-parametrically and control and intervention group will be compared using Wilcoxon's rank sum test.

#### Sample size and statistical power

The main outcome is knowledge, on which the sample size calculation is based. The knowledge questionnaire includes 8 items. Each item is rated right/wrong or not completed, which will be awarded with 0 or 1 point. Result of the knowledge test therefore cover 0-8 points. The statistical power using primarily Wilcoxon's rank sum test was estimated roughly using the power calculation of the corresponding t-test assuming approximate normal distribution. Nothing was known about the distribution of the ordinal outcome. A sample size of 527 evaluable participants per group will be enough to detect a difference of 0.20 σ of the expected values between intervention and control group by 90% power using a significance level of α = 5% and a 2-sided test (σ = common standard deviation on both groups). In view of the planned multiple regression analysis and the secondary analysis of the ordinal outcome a relatively large sample size was planned.

#### Ethical considerations

Participation is based on informed consent in accordance with the Declaration of Helsinki. All participants give their informed consent for participation in the study in an internet formulary.

The data security official of the German Diabetes Centre approved the study on 16/01/2008 and the amended protocol on 02/08/2009.

The Ethics Committee of the University of Düsseldorf approved the design of the study on 28/02/2008 and the amended protocol on 07/08/2009, reference number: 3020.

## Discussion

Diabetes prevention is currently being widely discussed. An evidence-based processing of the available evidence is therefore of great relevance and can be widely used.

In health services research patient-oriented questions and information transfer, information presentation and evaluation are increasingly playing a central role [34]. Thus, a thorough review of the project outcomes is of great interest, also from a scientific perspective.

Health information is increasingly available on the internet. An online information is therefore of major practical importance [35]. With the initial establishment of an evidence-based information to a "pre-diabetic" situation and primary prevention of diabetes and its use as an online-based evidence-based information, the project is also from a international scientific point of view a methodological challenge and markedly innovative. Web-based systems are not likely to achieve representative results for the population. Potential advantages lie in new opportunities for evaluation and possibly in a high cost effectiveness of the method. The design of this study may act as prototype for other web-based prevention information and their evaluation. Concerning potential long-term consequences of the intervention, no statements are possible at this time.

## Competing interests

The authors declare that they have no competing interests.

## Authors' contributions

This study was conceived and designed by all authors. JG contributed to the first and final draft of the manuscript. AI (guarantor) is the project leader and developed conception and design of the study. BH performs the statistical analysis. GG contributes biometric/statistical expertise. HM (guarantor of the online assessment) and DC contributed mainly to the development of the online process. FV contributed to study design and methodology and facilitated the online process. GM and AS developed and piloted the evidence based patient information. WR, BN and MR contributed clinical expertise, and AM contributed expertise in the field of socioeconomic aspects. CO is responsible for monitoring of the study quality. All authors reviewed the manuscript and made substantial contributions to subsequent drafts, and approved the final manuscript.

## Pre-publication history

The pre-publication history for this paper can be accessed here:

http://www.biomedcentral.com/1471-2458/10/15/prepub
